# Intrahepatic and Intrasplenic Pancreatic Pseudocysts Following Acute Pancreatitis: A Case Report

**DOI:** 10.7759/cureus.116024

**Published:** 2026-09-09

**Authors:** Sona Maghakyan, Tavros Tepanyan, Areg Petrosyan, Vahe Hakobyan, Andranik Aleksanyan

**Affiliations:** 1 Department of Internal Medicine, Mikaelyan Institute of Surgery, Yerevan, ARM; 2 Department of Surgery N1, Yerevan State Medical University After Mkhitar Heratsi, Yerevan, ARM; 3 Department of General Surgery, Mikaelyan Institute of Surgery, Yerevan, ARM

**Keywords:** alcohol-induced pancreatitis, hepatic pseudocyst, pancreatic pseudocyst (ppc), rare complications of pancreatitis, splenic pseudocyst

## Abstract

Pancreatic pseudocysts are common local complications of acute pancreatitis; however, intrahepatic and intrasplenic localization is exceptionally rare. The simultaneous occurrence of pancreatic pseudocysts in both the liver and spleen has been described in only a limited number of cases.

We present the case of a 64-year-old man with alcohol-related pancreatitis who developed multiple pancreatic pseudocysts involving the liver, spleen, left subphrenic space, lesser omentum, and falciform ligament. Contrast-enhanced computed tomography demonstrated large intrahepatic pseudocysts in segments II and III of the liver and a large intrasplenic pseudocyst. The patient was initially managed conservatively, followed by percutaneous drainage of the intrahepatic and left subphrenic pseudocysts. Due to the high risk of hemorrhage, the intrasplenic pseudocyst was not treated by percutaneous drainage. Progressive enlargement of the splenic pseudocyst necessitated open surgical drainage. The postoperative course was complicated by a pancreatic fistula, which resolved with conservative management within two months.

This case highlights the rarity of simultaneous intrahepatic and intrasplenic pancreatic pseudocysts and demonstrates that management should be individualized according to the location of the pseudocysts and the associated procedural risks. Percutaneous drainage may be effective for intrahepatic collections, whereas surgical intervention remains an important treatment option for selected splenic pseudocysts.

## Introduction

Pancreatic pseudocysts are among the most common local complications of acute pancreatitis. According to the revised Atlanta Classification (2012), a pancreatic pseudocyst is an encapsulated, well-defined collection of pancreatic fluid with little or no necrotic material, surrounded by a well-defined inflammatory wall, that usually develops more than four weeks after the onset of interstitial edematous pancreatitis [1].

Pancreatic pseudocysts are most commonly located adjacent to the pancreas, particularly in the pancreatic head and body. However, pancreatic fluid collections may extend into extrapancreatic locations depending on the site of pancreatic duct disruption and the anatomical pathway of enzyme leakage. Reported unusual locations include the mediastinum, pleural cavity, pelvis, liver, and spleen [2].

Extrapancreatic pseudocysts are relatively uncommon, occurring in approximately 20% of patients with pancreatic pseudocysts. The final location of these collections depends on the direction of pancreatic fluid propagation through anatomical planes [3].

Intrahepatic pancreatic pseudocysts are an exceptionally rare manifestation of pancreatitis. They are usually associated with acute pancreatitis, more commonly involve the left hepatic lobe, and may require percutaneous drainage or surgical treatment depending on their clinical characteristics [4].

Splenic involvement by pancreatic pseudocysts is also an uncommon complication of pancreatitis. Due to the risk of hemorrhage associated with splenic vascular structures, the optimal treatment approach remains controversial and should be individualized according to the patient's condition and anatomical findings [5].

We present the case of a patient with alcohol-related pancreatitis who developed multiple pancreatic pseudocysts involving both the liver and spleen, in addition to the left subphrenic space, lesser omentum, and falciform ligament. Although pancreatic pseudocysts with hepatic and splenic involvement have been reported sporadically in the literature [6,7], simultaneous intrahepatic and intrasplenic pseudocysts remain an exceptionally uncommon presentation.

## Case presentation

A 64-year-old man with a history of recurrent acute pancreatitis was admitted to our hospital with progressively worsening abdominal pain and nausea. His first episode of pancreatitis had been diagnosed approximately five years earlier, and he had experienced two additional episodes subsequently. There was no documented diagnosis of chronic pancreatitis. The current admission represented a new episode of acute pancreatitis, with symptoms beginning approximately 2-3 days before admission and progressively worsening during this period. The patient presented to the hospital because of the increasing severity and persistence of his abdominal pain, accompanied by nausea.

Laboratory investigations on admission revealed leukocytosis, with a white blood cell count of 18,580/mm³ (reference range, 3,500-10,000/mm³). The hemoglobin level was 140 g/L (reference range, 115-152 g/L). Serum amylase was elevated at 168 IU/L (reference range, <100 IU/L). Liver function tests showed an aspartate aminotransferase (AST) level of 4 IU/L (reference range, <37 IU/L) and an alanine aminotransferase (ALT) level of 14 IU/L (reference range, <41 IU/L).

The patient remained clinically stable during the initial conservative management, with no documented persistent organ failure or requirement for intensive care or organ-supportive therapy. Based on the clinical course, the current episode was considered mild acute pancreatitis according to the revised Atlanta Classification.

Contrast-enhanced computed tomography (CT) was performed on the day of admission and demonstrated inflammatory changes involving the pancreatic tail with extension into adjacent anatomical compartments. Multiple pancreatic and extrapancreatic fluid collections were identified, including collections involving the liver, spleen, left subphrenic space, lesser omentum, and falciform ligament. A well-defined cystic collection originating from the pancreatic tail extended toward the left subphrenic space. A large intraparenchymal splenic cystic lesion, measuring approximately 10 × 8 cm, with an average attenuation of 10 Hounsfield units (HU), was identified (Figure 1). The lesion was predominantly fluid-filled, without reported intralesional gas or definite solid necrotic debris. Another pancreatic fluid collection extended superiorly toward the liver, resulting in the formation of two large intrahepatic cystic lesions involving hepatic segments II and III, measuring 11 × 6 cm and 9 × 4 cm, respectively (Figure 1). No definite solid necrotic debris or intralesional gas was reported within these collections on the available CT images. 

**Figure 1:**
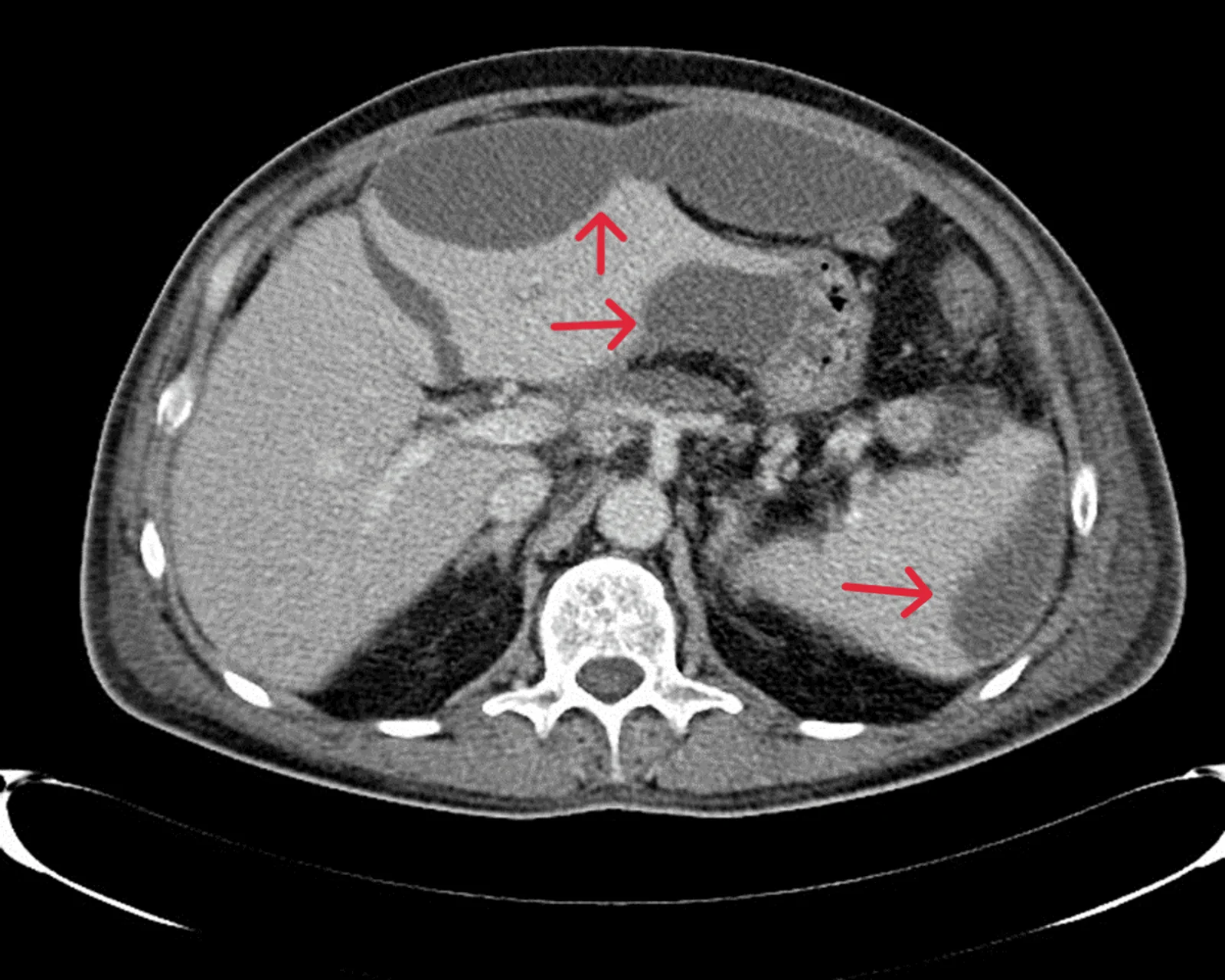
Contrast-enhanced computed tomography demonstrating multiple extrapancreatic pancreatic pseudocysts. A large intrasplenic pseudocyst is seen within the splenic parenchyma, with additional intrahepatic pseudocysts involving hepatic segments II and III and extension from the pancreatic tail toward adjacent anatomical compartments.

Another pancreatic fluid collection extended superiorly from the pancreatic tail toward the left subphrenic region and liver. Two large cystic lesions were identified within hepatic segments II and III, measuring 11 × 6 cm and 9 × 4 cm, respectively. These lesions were in close anatomical association with the adjacent extrapancreatic pancreatic fluid collection, supporting their interpretation as intrahepatic pancreatic fluid collections (Figure 1). Additional cystic collections were observed adjacent to the stomach, within the lesser omentum, and along the falciform ligament, indicating extensive extrapancreatic dissemination of pancreatic fluid.

Because the patient was clinically stable, initial conservative management was undertaken with close clinical and radiological follow-up. During follow-up, the intrahepatic and left subphrenic collections remained persistently large despite conservative management. There was no documented fever or clinical evidence of infection, and no specific complication such as gastric compression was reported. Image-guided percutaneous catheter drainage was therefore performed on hospital day 18 based primarily on the persistent size and anatomical location of the collections.

Follow-up CT demonstrated a significant reduction in the size of the drained intrahepatic and left subphrenic collections (Figure 2).

**Figure 2:**
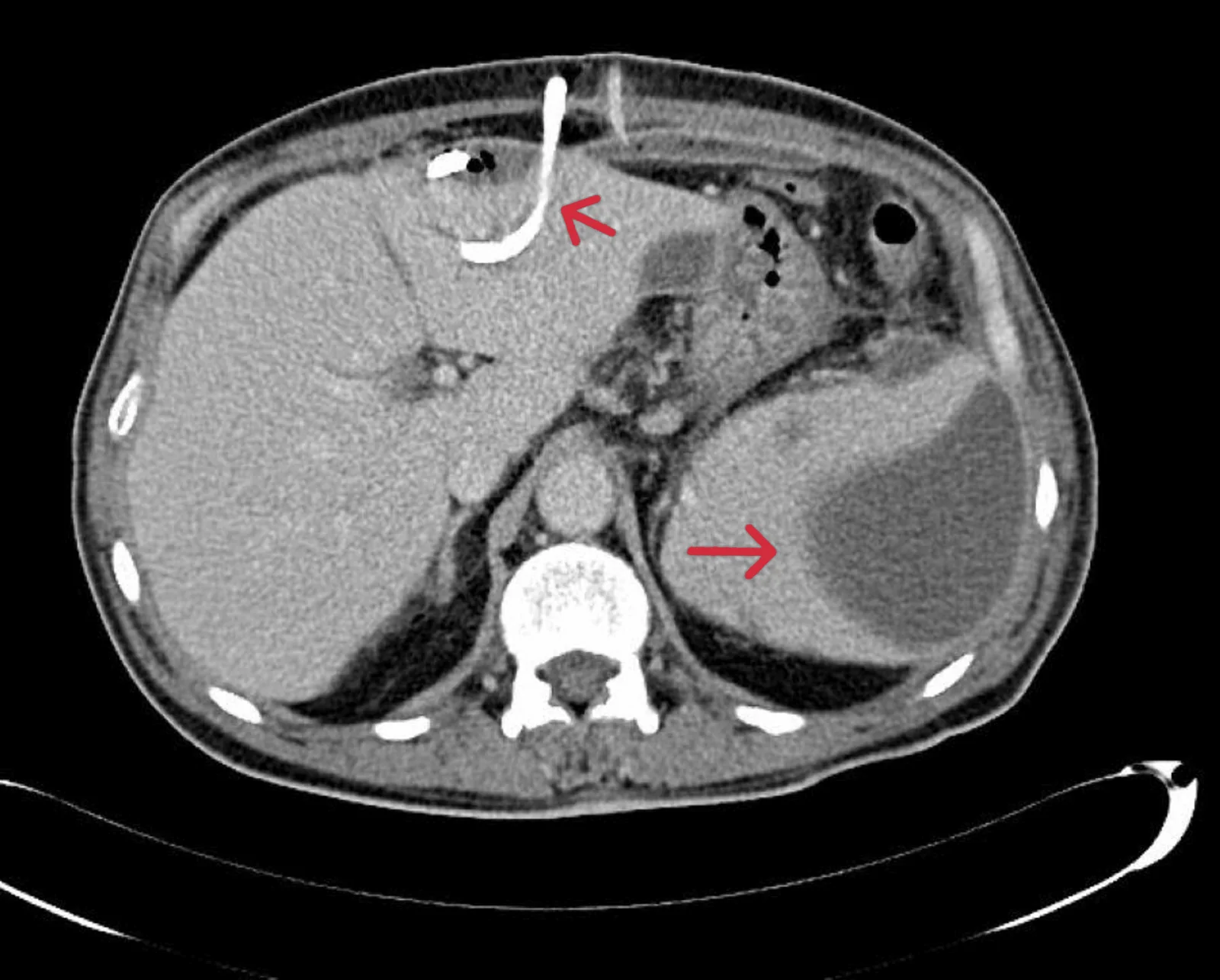
Follow-up CT scan after percutaneous drainage demonstrating interval changes in the previously identified fluid collections.

However, the intrasplenic pseudocyst showed no significant reduction in size. Percutaneous drainage of the splenic collection was not performed because of the increased risk of hemorrhage associated with the vascular anatomy of the spleen.

During subsequent follow-up, progressive enlargement of the intrasplenic collection was observed, accompanied by an increase in left pleural effusion. Due to the persistence and progression of the splenic collection, open surgical drainage was performed through a median laparotomy approximately one month after admission. The spleen was preserved, and neither splenectomy nor partial splenectomy was required.

Postoperative CT demonstrated a marked decrease in the size of the cystic collections (Figure 3).

**Figure 3:**
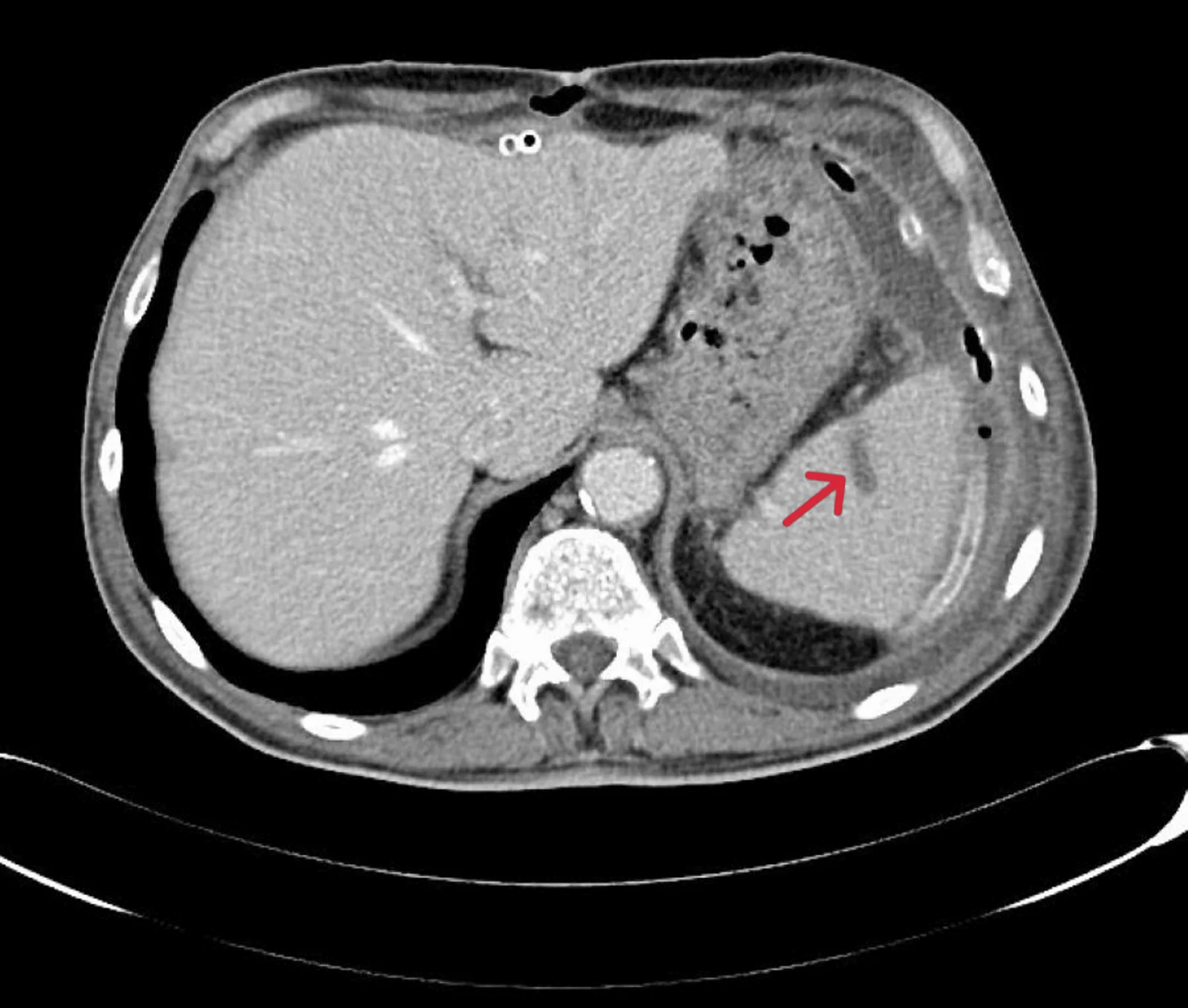
Postoperative contrast-enhanced CT scan demonstrating reduction in the size of the cystic lesions after surgical drainage of the intrasplenic collection.

The postoperative course was complicated by a clinically relevant postoperative pancreatic fistula, which was managed conservatively. No reoperation or additional invasive intervention was required. The patient remained clinically stable, and the fistula resolved completely within approximately two months.

## Discussion

Pancreatic pseudocysts are among the most common local complications of acute pancreatitis. They develop as a consequence of pancreatic fluid leakage and encapsulation by fibrous and granulation tissue. According to the revised Atlanta Classification (2012), pancreatic pseudocysts are well-defined encapsulated fluid collections that usually develop more than four weeks after the onset of interstitial edematous pancreatitis and contain little or no solid necrotic material [1]. Most pancreatic pseudocysts are located adjacent to the pancreas, particularly around the pancreatic head and body; however, extension into extrapancreatic locations may occur depending on the pathway of pancreatic fluid dissemination [3].

Extrapancreatic pancreatic pseudocysts are uncommon and may involve unusual anatomical locations, including the mediastinum, pleural cavity, liver, spleen, and other adjacent structures. The mechanism of extension is related to the spread of pancreatic secretions through anatomical planes and areas of least resistance [2]. Although uncommon, these atypical localizations may create diagnostic and therapeutic challenges.

Intrahepatic pancreatic pseudocysts represent a rare complication of pancreatitis. Previous reports have described hepatic involvement mainly following acute pancreatitis, with a tendency toward localization in the left hepatic lobe. The diagnosis of an intrahepatic pancreatic fluid collection is primarily supported by the clinical context of pancreatitis and characteristic imaging findings demonstrating a cystic lesion in anatomical association with pancreatic fluid collections. Important imaging differentials include pyogenic or amebic liver abscesses, echinococcal cysts, and biliary cystic neoplasms. In the present case, cyst fluid biochemical analysis, including amylase or lipase measurement, was not available; therefore, the pancreatic origin was inferred from the overall clinical and radiological findings. Treatment options include conservative management, percutaneous drainage, and surgical intervention, depending on the size, symptoms, anatomical location, and evolution of the collection [4]. A systematic review by Guesmi et al. emphasized the rarity of intrahepatic pancreatic pseudocysts and the need for individualized therapeutic approaches [8]. Several case reports have further described different patterns of hepatic involvement, including left lobe localization and rupture into the liver parenchyma [9,10]. EUS-guided transmural drainage, including cystogastrostomy or cystoduodenostomy, is an established option for appropriately located pancreatic pseudocysts; however, its applicability may be limited in unusual intrahepatic or intraparenchymal locations [11].

Splenic involvement secondary to acute pancreatitis is rare (approximately 2%). The close anatomical relationship between the pancreatic tail and splenic hilum allows pancreatic fluid collections to extend into the spleen. Management should be individualized according to the patient's clinical condition, size and anatomical characteristics of the collection, and the risks associated with intervention. Potential treatment options include observation in selected stable patients, percutaneous drainage, endoscopic approaches when anatomically feasible, and surgical intervention, including splenectomy or distal pancreatectomy in selected cases [5,11].

In the present case, a 64-year-old male with alcohol-related pancreatitis developed multiple extrapancreatic fluid collections involving the liver, spleen, left subphrenic space, lesser omentum, and falciform ligament. Percutaneous drainage was performed for the intrahepatic and left subphrenic collections, while drainage of the intrasplenic collection was avoided initially because of the high risk of hemorrhage. Due to persistent splenic involvement, open surgical drainage was subsequently performed. The postoperative pancreatic fistula resolved with conservative management within two months.

This case emphasizes that management of pancreatic pseudocysts with unusual extrapancreatic localization should be individualized according to anatomical location, clinical course, and the potential risks associated with each intervention. Although intrahepatic and intrasplenic pancreatic pseudocysts are rare, they should be considered in patients with pancreatitis who develop atypical cystic lesions on imaging.

Although the diagnosis was supported by the overall clinical and radiological findings, direct communication between the pancreatic duct or pancreatic tail collection and the splenic lesion was not demonstrated on the available imaging. In addition, cyst fluid biochemical analysis, including amylase measurement, was not available. The available radiological records provided two-dimensional measurements of the collections but did not include standardized three-dimensional volumetric measurements or detailed characterization of wall thickness, wall enhancement, or internal septations for all lesions. Therefore, retrospective calculation of the true fluid volume was not possible. The pancreatic origin of the splenic collection was inferred from the clinical context, anatomical relationship, and imaging findings. 

The postoperative pancreatic fistula may have resulted from persistent pancreatic duct disruption or an occult communication between the pancreatic tail and the cystic collection, allowing pancreatic secretions to drain through the surgical tract. As direct ductal communication was not demonstrated, this represents a possible rather than confirmed mechanism.

This case illustrates that the anatomical location of an extrapancreatic pancreatic collection may be an important determinant of treatment strategy. The intrahepatic collections were amenable to percutaneous drainage and subsequently decreased in size, whereas percutaneous access to the intraparenchymal splenic collection was considered potentially hazardous because of the risk of vascular injury and hemorrhage. The initial observation of the splenic collection was therefore changed to surgical drainage after progressive enlargement during follow-up, accompanied by an increase in the left pleural effusion. Previous reports have described successful conservative, percutaneous, and surgical management of intrahepatic and intrasplenic pancreatic collections, supporting an individualized approach based on anatomical location, clinical course, and procedural risk [3-5,8,9].

## Conclusions

Intrahepatic and intrasplenic pancreatic pseudocysts are rare extrapancreatic complications of pancreatitis that may present diagnostic and therapeutic challenges. Careful evaluation of imaging findings is important for establishing the diagnosis and planning management. In the present case, percutaneous drainage was effective for the intrahepatic and left subphrenic collections, whereas surgical drainage was ultimately required for the progressively enlarging intrasplenic collection. The postoperative pancreatic fistula resolved with conservative management. This case illustrates that management of such unusual pancreatic fluid collections should be individualized according to their anatomical location, clinical course, and the risks associated with intervention.
